# A Brief Biofeedback Training, Integrated with Breathing and Relaxation Exercises, in Treating Tinnitus Disorders within Routine Medical Care

**DOI:** 10.1007/s10484-025-09694-1

**Published:** 2025-02-06

**Authors:** Chiara Buizza, Elena Franco, Alberto Ghilardi, Herald Cela

**Affiliations:** 1 Department of Clinical and Experimental Sciences, University of Brescia, Viale Europa 11, Brescia, Italy; 2 Medical Center San Francesco, Via Zadei 16, Brescia, Italy; 3 Department of Psychology, University of Graz, Universitätsplatz 2, Graz, 8010 Austria

**Keywords:** Tinnitus, Biofeedback, Relaxation Training, Breathing Exercises, Effectiveness, Handicap

## Abstract

Tinnitus, a distressing condition, significantly impacts psycho-social functioning. While medical interventions have been the norm for treating tinnitus, few studies have explored the efficacy of psychological treatments and their enduring effects. This study aims to evaluate the effectiveness of a brief biofeedback training program in alleviating perceived tinnitus handicap severity over a 3-month follow-up period. Engaging 431 tinnitus outpatients from a medical center, the study allocated the control group to treatment as usual, involving monthly visits to an otolaryngologist and specific pharmacological interventions. Concurrently, the experimental group participated in a brief biofeedback training, using Procomp Infinity by Thought Technology Ltd., an eight-channel computer-operated encoder, integrated with breathing and relaxation exercises. No biofeedback booster sessions were provided to the experimental group during the 3-month follow-up period. Changes in tinnitus severity were assessed using the Tinnitus Handicap Inventory (THI). The analysis, conducted via ANCOVA, demonstrated that biofeedback, integrated with relaxation training and breathing exercises, had a positive impact on both the follow-up THI total score and its three sub-scales (Functional, Emotional, Catastrophic). Notably, the experimental group displayed reduced psycho-physiological parameters in all aspects compared to their baseline measurements at 3-month follow-up. This study’s findings underline the effectiveness of non-pharmacological intervention in treating tinnitus. It had a positive impact on the emotional, functional, and physical dimensions of daily life affected by tinnitus.

## Introduction

Tinnitus is a clinical condition characterized by the perception of sound in the absence of a corresponding acoustic source (Baguley et al., 2013) affecting 10–15% (Shargorodsky et al., 2010) of the population. Its prevalence is higher among the elderly (12% after age 60) compared to young adults (5% in the 20–30 age groups), yet it can manifest at any age (Dobie, 2003).

The etiopathogenesis of tinnitus remains still an element of discussion, characterized by multiple causative factors and an evident lack of consensus. Goodhill’s classification divides tinnitus into subjective and objective types (Filipo, 1973). Subjective tinnitus, perceivable only by the patient, arises from heterogeneous etiopathogenetic factors such as organic-functional disorders along the “acoustic path”, and stress. Objective tinnitus involves noise originating outside the ear, perceived by an undamaged hearing system. According to Goodhill’s classification, objective tinnitus includes cases of muscular origin (e.g., ear muscle spasms, masticatory, cervical) and cases of vascular origin (e.g., carotid and arteriovenous aneurysms at the base of the skull, stenosis and thrombosis of the vertebral artery or of the basic).

Chronic tinnitus, lasting for six months or more, rarely experiences spontaneous remission. While some individuals with tinnitus manage it effectively, many suffer from highly disabling ear noises, often accompanied by comorbidities such as depression, demoralization, social withdrawal, headaches, and sweating (Rief et al., 2005). These challenges can lead to significant distress, with an important consequent impairment of psycho-social functioning (Brüggemann et al., 2016). Indeed, tinnitus creates a profoundly disabling condition that impacts patients’ psychological and emotional well-being, relationships, sleep patterns, work behavior, and exacerbates anxiety, thereby negatively interfering with their quality of life (McKenna et al., 2014; Zirke et al., 2016).

Tinnitus is a complex and multidimensional phenomenon that extends beyond the auditory realm. For this reason, a multidisciplinary approach is necessary, involving different professionals such as otolaryngologists, vascular and neurologists, psychologists. Treatment strategies include not only a multidisciplinary approach, but also different levels of therapy (home-based, outpatient or hospitalization) depending on the severity of the condition (Bardsiri et al., 2018). The intricate interplay of multiple etiological and pathological causes often pose challenges to effective resolution, making the prognosis for tinnitus a complex and variable matter.

Until now, the treatment of tinnitus has primarily focused on medical interventions, with an emphasis on organic therapies. However, symptoms of distress, anxiety, or depression frequently coexist with tinnitus, often overshadowing the primary pathological condition (Bhatt et al., 2017). Despite this, psychological treatment is often not offered to patients. Among psychological treatments, biofeedback can be an effective technique given that it has long been used for stress management or for the treating disorders in which stress plays a significant role (Buizza et al., 2023; Goessl et al., 2017; Lehrer et al., 2013; MacKinnon et al., 2013; Ratanasiripong et al., 2015; Sherlin et al., 2009). Indeed, the mechanisms underlying the etiology of tinnitus highlight a vicious circle wherein stress contributes to tinnitus and is also a consequence of its disabling nature. Biofeedback has been shown to positively affect self-rated stress and discomfort in people suffering from subjective tinnitus (Heinecke et al., 2008; Lindberg et al., 1987; Meckley Kutyana, 2015). Several studies have demonstrated the effectiveness of biofeedback in reducing symptoms, anxiety and depression commonly associated with tinnitus (Heinecke et al., 2009; Rief et al., 2005; Weise et al., 2008). However, these studies (Heinecke et al., 2008; Weise et al., 2008; Rief, 2005) utilised latest generation biofeedback tools (Flexcomp Infinity with Biograph Infinity software) primarily focusing on measuring and analyzing skin conductance and muscle reactivity. None of these studies examined respiratory parameters, with only one study (Meckley Kutyana et al., 2015) evaluating the impact of heart rate variability, albeit on a small sample (three case studies). Although these studies have shown that biofeedback can be an effective component of a multidisciplinary approach to treating tinnitus, many of them lacked follow-up assessments and required intensive biofeedback treatment (Landis & Landis, 1992; Heinecke et al., 2009). Additionally, some studies involved small sample sizes (House, 1978; Haralambous et al., 1987).

This feasibility study aimed to assess the therapeutic effectiveness of a brief biofeedback training, integrated with breathing and relaxation exercises, in treating tinnitus disorders within the routine of a clinical service.The goal was to mitigate emotional, functional, and catastrophic disability over a 3-month follow-up period, thus enhancing the well-being of individuals afflicted by tinnitus.

## Materials and Methods

### Study Design

This study involved two groups of outpatients: the control group received treatment as usual, consisting of one monthly visit with an otolaryngologist and pharmacological treatment specific for tinnitus (cinnarizine, corticosteroids, vitamin supplements), while the experimental group participated in an additional brief biofeedback training program, integrated with breathing and relaxation exercises. In the experimental group, pharmacological therapy was administered solely in the phase preceding the commencement of biofeedback, in order to stabilize any acute phase associated with hearing loss onset or exacerbation. During the 3-month follow-up, the experimental group did not receive any biofeedback boosting sessions.

### Participants

The medical center where the study was conducted is an innovative private outpatient healthcare facility. Otolaryngologists participating in the study were informed of the option to refer tinnitus patients for potential biofeedback treatment. Patients were offered an opportunity to become part of the experimental group. Patients paid for the biofeedback sessions, and medications prescribed by the otolaryngologist were purchased at pharmacies in accordance with Italian regulations. Patients had to meet the following inclusion criteria: diagnosis of cochlear hydrope or vascular cochleopathy, aged 18 years or older, pharmacological stabilization provided only immediately before commencing biofeedback to address acute phases with hearing loss onset or exacerbation, and sufficient proficiency in their native language to complete the questionnaire. Individuals with tinnitus caused by substance abuse were excluded from the study.

A total of 431 tinnitus patients, ranging in age from 18 to 89 years, were consecutively enrolled between May 2019 to December 2021. Due to the pragmatic nature of the trial conducted under routine circumstances, randomization was not feasible. A natural control group was utilized, involving patients who met the criteria for participation and who were available to attend the paid biofeedback sessions. They were placed on a waiting list due to space constraints. Because the limited availability of biofeedback therapists, a total of 217 patients could be treated at the medical center over a two-year period.

The study adhered to the principles expressed in the Declaration of Helsinki and received approval from the Director of the tinnitus treatment center. All the participants provided written informed consent prior to the start of the study and they retained the liberty to withdraw from the study at any point.

### Measures

#### Socio-Demographic Assessment Form

Participants were requested to fill out a socio-demographic and clinical assessment form, providing details such as gender, age, employment status, diagnosis, symptoms onset, previous hospitalizations, and familiarity with tinnitus (i.e., presence of family members who suffered from tinnitus).

#### Tinnitus Handicap Inventory (THI)

The THI is a self-report questionnaire designed to evaluate perceived tinnitus handicap severity (Newman et al., 1996; Monzani et al., 2008). It consists of 25 items, with each item offering a choice between three answers: *yes* (4 points), *sometimes* (2 points), *no* (0 points). The overall score ranges from 0 (no disability) to 100 (maximum self-perceived disability). The score ranges are categorized as follows (Newman & Jacobson, 1998):

- Scores 0 to 16: “slight or no handicap”. Tinnitus heard only in quiet settings, easily masked without affecting sleep or daily activities.
- Scores 18 to 36: “mild handicap”. Tinnitus masked by environmental sounds, easily ignored during activities, occasional interference with sleep and non-daily activities.
- Scores 38 to 56: “moderate handicap”. Tinnitus noticeable despite background noise, although daily activities may still be performed.
- Scores 58 to 76: “severe handicap”. Tinnitus nearly always heard, rarely masked, disturbs sleep and daily activities, affects quiet tasks.
- Scores 78 to 100: “catastrophic handicap”. Tinnitus always heard, disrupts sleep and all activities.

The THI is composed of three sub-scales: *functional* (examining limitations due to tinnitus in mental, social, and physical functioning); *emotional* (assessing affective responses to tinnitus, like anger, frustration, depression, and anxiety); and *catastrophic* (exploring the most severe reactions to tinnitus, such as loss of control, inability to escape from tinnitus, and fear of having a terrible disease).The Italian version of the THI showed a robust internal consistency, as indicated by a Cronbach’s alpha of 0.91 for the THI total score, and 0.85, 0.86, 0.63 for the functional, emotional and catastrophic sub-scales respectively (Monzani et al., 2008). In our sample, the Cronbach’s alpha values were 0.75 for the total score, 0.48 for the functional sub-scale, 0.58 for the emotional sub-scale, and 0.33 for the catastrophic sub-scales.

#### Biofeedback Treatment, Relaxation Training and Breathing Exercises

The biofeedback treatment was administered by psychologists specialized in psychotherapy, who had undergone specific training in biofeedback usage. Some reference manuals were used, such as those by Schwartz and Andrasik (2017) and Khazan (2018).

The biofeedback treatment consisted of a variable number of sessions, ranging from a minimum of 1 to a maximum of 5 per month, with each session lasting approximately 60 min. During these sessions, participants engaged in biofeedback exercises aimed at regulating parameters such as respiratory frequency, heart rate, and muscle tension. Between sessions, patients were given exercises to improve breathing, relax muscles, and manage their tinnitus-related symptoms. At the beginning of each new biofeedback session, the homework assignments and any difficulties encountered were reviewed together with the patient.

All psycho-physiological data were recorded using Procomp Infinity by Thought Technology Ltd., an eight-channel computer-operated encoder. The software used was BioGraph Infinitiy (version 6.2.0.). Muscle activity was assessed for various regions, including the forehead (frontalis region), jaws (masseter, bilateral), and neck (sternocleidomastoids, bilateral). Of particular importance for tinnitus patients, electromyography (EMG) electrodes were placed over the anterolateral muscles of the sternocleidomastoid neck. Following the recommendations of Cram (1990), the electrodes were positioned parallel to muscle fibers to maximize sensitivity and selectivity. T9503M MyoScan EMG sensors, pre-amplified, were employed, possessing an input impedance greater than 10 KOhm and an active range spanning from 10 to 500 Hz, as outlined by Cacioppo et al. (2000), and by Konrad (2005). Dry T340M Triodes were utilized as electrodes. A conductive gel was used between the electrode and the skin. The raw EMG signal was converted to root mean square (RMS) using the non-sliding-window algorithm with an averaging factor of 10 and a time period of 1s. A tolerance for artifact was incorporated to facilitate measurement of slow muscle activity.

Biofeedback leverages the electrical aspects of muscle contraction. Muscle contraction results from the more or less synchronous activation of the many muscle fibers that make up a muscle. These muscle fibers are triggered by electrical signals transmitted by cells called ‘motor units.’ The contraction of a muscle corresponds to the combined electrical activity within these fibers. An EMG device measures an electrical correlate of muscle contraction, providing output in electrical units (microvolts) (Schwartz & Andrasik, 2017).

Skin conductance level (SCL) was recorded using the SA9309M Skin Conductance Flex/Pro, which featured a signal range of 0 to 30 µS. A skin conductance device applies a very small electrical voltage to the skin, typically on the volar surface of the fingers or the palmar surface of the hand, where there are many sweat glands. It measures the amount of electrical current that the skin allows to pass. The magnitude of this current indicates the level of skin sweatiness and is expressed in units of electrical conductance called microsiemens (Schwartz & Andrasik, 2017). It is noteworthy that skin conductance was not measured for 20 patients (9.2% of the sample) due to various medical conditions such as pacemakers, epilepsy, heart disease, or pregnancy. This omission was due to the potential risks associated with the sensor’s discharge in these specific cases (Shaffer & Meehan, 2020).

Finger temperature measurements were acquired through the SA9310M sensor, with a signal range spanning from 12.5 to 40.5 °C. Prior to electrode attachment, the skin was cleaned with an antiseptic.

A biofeedback device cannot directly measure the changing diameter of peripheral blood vessels. However, dilated vessels allow more warm blood to flow through than constricted vessels. As a result, the surrounding tissue tends to warm or cool as the vascular diameter increases or decreases, offering a reliable correlate of vascular diameter. The biofeedback device provides a readout in degrees Fahrenheit as an indirect indicator of peripheral vasoconstriction (Schwartz & Andrasik, 2017).

Respiratory and Heart Rate (HR) were measured with a breath sensor SA9311M and a Blood Volume Pulse (BVP) frequency sensor, alongside a plethysmography wristband SA9308M.

This device, a photoplethysmograph, monitors pulse and, with appropriate circuitry to average out the pulse, can provide an indication of relative blood volume, another correlate of vasoconstriction (Schwartz & Andrasik, 2017).

Psycho-physiological parameters, encompassing EMG (µV), respiratory rate (number of breaths per minute), SCL (µS), peripheral temperature, the bpm for BVP heart rate, and the low-frequency to high-frequency (lf/hf) ratio, were evaluated during THI administration.

For the experimental group, which received biofeedback training, an initial and final baseline of two minutes was established. The experimental group underwent respiratory biofeedback according to the following indications (Khazan, 2018).

- Explain the patient the physiology of breathing and over breathing.
- Foster awareness of breathing, assisting the patient in connecting with the emotions associated with effortless breathing.
- Teach slow and deep diaphragmatic breathing without feedback.
- Instruct controlled diaphragmatic breathing with a of 40:60 inhalation-to-exhalation ratio, with or without a pause between exhalation and the subsequent inhalation. In particular, the resonance frequency of the respiratory rhythm is identified, and diaphragmatic breathing is synchronized with this frequency. During training sessions, patients are taught to increase Heart Rate Variability (HRV) according to established protocols, such as the Heart Rate Variability Biofeedback Training protocol (Lehrer et al., 2013). These sessions facilitate the teaching of patients to breathe at the resonant frequency of their cardiovascular system, which optimizes respiratory effects on HR and baroreflex stimulation. Breathing at this frequency synchronizes HR fluctuations with respiration (heart rate increases with inhalation, decreases with exhalation), maximizing respiratory gas exchange efficiency (Vaschillo et al., 2004; Yasuma & Hayano, 2004). Patients are able to produce significant increases in HRV through biofeedback, facilitated by ‘resonance’ attributes of the cardiovascular system. HRV biofeedback, by stimulating the baroreflex mechanism, contributes to blood pressure control and emotional reactivity modulation. When blood pressure increases, baroreflex decreases HR, and viceversa (Lehrer et al., 2013; Vaschillo et al., 2006). This rhythm in HR fluctuations resonates when patients breathe at this specific frequency (Lehrer et al., 2013; Schwartz & Andrasik, 2017). Breathing at this frequency amplifies the baroreflex system, reinforcing cardiovascular stability and producing the beneficial effects of HRV biofeedback (Lehrer et al., 2013). Clear and accurate instructions were provided to the patients during the biofeedback sessions, aiming to optimize their utilization of the feedback signal provided by the biofeedback system. The goal was to help the patients gain awareness of their own physiological indices.
- Encourage practice of breathing exercises at home, for 20 min twice a day. Patients can use the second hand of a clock or downloaded resonant-frequency breathing rhythms from a computer. Patients are advised to be mindful of symptoms of hyperventilation and, if experienced, to revert to shallow and natural breathing.

Additionally, the experimental group underwent skin conductance and EMG biofeedback following these guidelines (Khazan, 2013): teach progressive muscle relaxation without feedback; introduce biofeedback using visual and audible cues linked to skin conductance and EMG muscle tension benchmarks; monitor skin conductance and muscle tension during Jacobson’s progressive muscle relaxation and monitor the ensuing changes in response; assign respiratory and muscle relaxation exercises to be performed at home to enhance awareness.

#### Follow-Up

Three months after completing the treatment, patients were re-evaluated by the therapists who had conducted the biofeedback, relaxation training and breathing exercises. The experimental group were assessed with EMG, respiratory rate, skin conductance, peripheral temperature, HR, and lf/hf parameters during THI administration, with an initial and final baseline of two minutes. A three-month follow-up was also conducted for the control group. In this instance, only the THI was re-administered.

#### Main Outcome Measurement

The main outcome of this study was to assess the effectiveness of a brief biofeedback training program, integrated with breathing and relaxation exercises, within a regular medical center for treating tinnitus. This assessment focused on the reduction of self-perceived disability, as measured by the THI questionnaire, at 3-month follow-up.

#### Statistical Analysis

Descriptive statistics, including means and standard deviations, were calculated to summarize the sociodemographic variables and questionnaire-based measures at baseline for each group. The comparability of baseline variables between the two groups was evaluated through independent samples t-tests or appropriate non-parametric tests. The correlation between continuous variables was computed using Spearman’s correlation coefficient.

To evaluate the effectiveness of the intervention on the THI total score and its subscales, four separate analysis of covariance (ANCOVA) models were performed. Each model analysed one dependent variable independently: the THI total score or one of its three sub-scale scores (Functional, Emotional, and Catastrophic). The experimental group was entered as a fixed factor, and baseline values of the corresponding outcome measure were included as covariates to account for pre-existing differences (Stevens, 2012; Tabachnick et al., 2013). By conducting independent models for each outcome, we ensured that the assumption of independence between dependent variables was maintained. ANCOVA was selected for its capability to adjust for the baseline differences between groups and reduce confounding effects, thus enabling a more precise evaluation of the intervention’s influence on the outcome variables (Stevens, 2012; Tabachnick et al., 2013). Assumptions of ANCOVA, such as normality and homogeneity of variances, were assessed. The assumption of homogeneity of regression slopes, which presumes that relationships between covariates and dependent variables are consistent across groups, was inspected via scatterplots and interaction analyses (Field, 2018; Osborne, 2013). For robustness assessment, bootstrap resampling with bias-corrected and accelerated (BCa) confidence intervals were employed for ANCOVA results (Efron & Tibshirani, 1993; Mooney et al., 1993). The bootstrap procedure entailed generating multiple resamples from the original data, computing ANCOVA for each resample, and estimating confidence intervals based on the bootstrap statistics distribution. This approach accounted for potential assumptions violations, offered more robust inferences, and allowed for a comprehensive evaluation of treatment’s effects stability (Efron & Tibshirani, 1993; Mooney et al., 1993).

In addition to questionnaire-based measures, physiological data (e.g., skin conductance) were exclusively collected for the experimental group, both pre and post-biofeedback. To gauge the biofeedback intervention’s influence on these physiological parameters, related samples analyses were conducted, permitting within-group comparisons of the pre- and post-treatment values.

Statistical analyses were conducted using SPSS 29.0 (IBM Corp., Armonk, NY). A significance level of α = 0.05 was employed to ascertain statistical significance.

## Results

### The Groups’ Baseline Characteristics

Table 1 displays the main characteristics of the two groups. The experimental group comprised 217 patients (128 males, 89 females), while the control group consisted of 214 patients (96 males, 118 females). A higher proportion of males was observed in the experimental group compared to the control group (χ² = 8.617; df = 1; *p* =.003, z-score = 2.9). The mean age was significantly higher in the experimental group than in the control group (t test = 2.036, *p* =.021; η_p_^2^ = 0.2). Additionally, a larger percentage of unemployed individuals was present in the experimental group compared to the control group (χ² = 4.780; df = 1; *p* =.029, z-score = 2.2).

**Table 1:** Comparison of sociodemographic and clinical characteristics of participants at baseline

	Experimental group (*N* = 217)	Control group (*N* = 214)	Statistical test	*p* value
*Age*,* mean (sd)*	54.0 (14.5)	49.8 (19.8)	2.498^a^	**< 0.006**
*Illness duration (year)*,* mean (sd)*	3.4 (4.7)	2.8 (2.1)	24.720^b^	0.073
*Illness severity THI*,* mean (sd)*				
Total score Functional sub-scale Emotional sub-scale Catastrophic sub-scale	46.8 (21.4) 19.4 (10.6) 16.1 (9.3) 11.4 (5.5)	48.7 (13.6) 22.6 (6.6) 15.5 (5.7) 10.6 (4.2)	-1.088^a^ 27.801^b^ 21.722^b^ 21.109^b^	NS **< 0.001** NS NS
*Sex*,* n (%)*				
Male Female	128 (59.0) 89 (41.0)	96 (44.8) 118 (55.2)	8.614^c^	**0.003**
*Employed*,* n (%)*				
Yes No	148 (68.2) 69 (31.8)	166 (77.6) 48 (22.4)	4.780^c^	**0.029**
*Diagnosis*,* n (%)*				
Cochlear hydrops Vascular cochleopathy	193 (88.9) 24 (11.1)	190 (88.7) 24 (11.3)	.003^c^	NS
*Previous hospitalizations*,* n (%)*				
Yes No	20 (9.2) 197 (90.8)	16 (7.5) 198 (92.5)	.426^c^	NS
*Familiarity for tinnitus*, *n (%)*				
Yes No Missing	92 (42.4) 125 (57.6) -	83 (38.8) 130 (60.7) 1 (0.5)	1.540^c^	NS
*Drugs therapy*,* n (%)* *Cinnarizine*				
Yes No	159 (73.3) 58 (26.7)	177 (82.7) 37 (17.3)	5.586^c^	**0.018**
*Corticosteroid*				
Yes No	64 (29.5) 153 (70.5)	120 (56.1) 94 (43.9)	31.117^c^	**< 0.001**
*Vitamin supplements*				
Yes No	81 (37.3) 136 (62.7)	137 (64.0) 77 (36.0)	30.709^c^	**< 0.001**
*Number of biofeedback sessions attended by patients*,* mean (sd)*	3.2 (1.5)	-	-	-

Note.^a^T-test; ^b^Mann-Whitney; ^c^Chi square; df = 1; NS = Not Significant

No significant differences emerged concerning illness duration, previous hospitalization, familiarity with tinnitus, and the predominant diagnosis, which was primarily cochlear hydrops in both groups (88.9% in the experimental group vs. 88.7% in the control group).

Regarding the THI total score, comparable results were observed between the two groups. However, in relation to the three sub-scales, a statistically significant difference was identified solely in the functional sub-scale, where the control group exhibited a significantly higher score than the experimental group (U = 27.801, *p* =.001; η_p_^2^ = 0.2).

Concerning drug therapy, the control group displayed higher use of cinnarizine (χ² = 5.586; df = 1; *p* =.018, z-score = 2.4), corticosteroids (χ² = 31.117; df = 1; *p* = < 0.001, z-score = 5.6), and vitamin supplements (χ² = 30.709; df = 1; *p* = < 0.001, z-score = 5.5).

### Differences between the Two Groups at 3-month Follow-Up

All patients provided follow-up data. Prior to conducting the analysis, we visually examined the normality of the dependent variables (THI score and its sub-scales) using histograms. The distribution of the THI scores appeared to be approximately normal. Additionally, we assessed the homogeneity of the regression slopes assumption by inspecting scatterplots and evaluating the significance of the interaction term between the intervention group and the baseline scores. Although the scatterplot regression lines were similar, the interaction term for the covariate and the treatment was significant in all models, thereby violating the assumption of homogeneity of regression slopes. To address this, we employed robust methods with bootstrapping to obtain more reliable confidence intervals around the mean. Table 2 displays the differences between the two groups in the THI average scores. The statistical analysis showed differences in the THI total score between the two groups at follow-up (F(1,428) = 1171.20, *p* <.001, η_p_² = 0.73). The experimental group displayed a significantly lower THI total score (Adj. mean = 19.28, BCa 95% CI [17.90, 20.94]) compared to the control group (Adj. mean = 46.75, BCa 95% CI [45.13, 48.40]). The mean difference between the experimental and control groups was statistically significant at 27.47 (SE = 0.83, t = -33.20, *p* <.001, BCa 95% CI [-29.10, -25.84], η_p_² = 0.72). The experimental group’s score indicated a mild tinnitus level post-treatment, easily masked by environmental sounds and activities. In contrast, the control group exhibited a moderate tinnitus level, noticeable even amid background noise, albeit not significantly affecting daily activities. Subsequent models were fitted on the subscales of THI.

**Table 2:** Differences between the two groups in the THI average scores (baseline vs. follow-up)

	Baseline				Follow-up				
	Experimental group (*N* = 217)	Control group *N* = 214)	Statistical test	*p* value	Experimental group (*N* = 217)	Control group (*N* = 214)	Statistical test (F)	*p* value	ES (η_p_^2^)
*THI*,* mean (sd)*									
Total score Functional sub-scale Emotional sub-scale Catastrophic sub-scale	46.8 (21.4) 19.4 (10.6) 16.1 (9.3) 11.4 (5.5)	48.7 (13.6) 22.6 (6.6) 15.5 (5.7) 10.6 (4.2)	-1.088^a^ 27.801^b^ 21.722^b^ 21.109^b^	NS **0.001** NS NS	18.75 (13.02) 8.05 (6.68) 6.58 (5.63) 4.12 (4.03)	47.30 (13.20) 19.42 (5.54) 17.43 (6.63) 10.44 (4.15)	1.171.20 398.14 527.49 342.18	**< 0.001** **< 0.001** **< 0.001** **< 0.001**	0.73 0.48 0.55 0.44

Note. Raw means at for THI at follow-up, between experimental and control groups. F, p, partial eta squared represent the effect for group while considering the baseline in the ANCOVA model. ^a^T-test; ^b^Mann-Whitney

Differences between the two groups at follow-up were also found on the functional sub-scale (F(1,428) = 398.14, *p* <.001, η_p_² = 0.48). The experimental group exhibited a significantly lower score on the functional sub-scale (Adj. mean = 8.80, BCa 95% CI [8.01, 9.70]) compared to the control group (Adj. mean = 18.65, BCa 95% CI [17.83, 19.44]). The mean difference between the experimental and control groups was statistically significant at -9.85 (SE = 0.54, t = -18.14, *p* <.001, BCa 95% CI [-10.92, -8.78], η_p_² = 0.43).

Differences between the two groups at follow-up were found on the emotional sub-scale (F(1,428) = 527.49, *p* <.001, η_p_² = 0.55). The experimental group demonstrated significantly lower scores on the emotional sub-scale (Adj. mean = 6.42, BCa 95% CI [5.79, 7.10]) compared to the control group (Adj. mean = 17.59, BCa 95% CI [16.70, 18.50]). The mean difference between the experimental and control groups was statistically significant at -11.17 (SE = 0.48, t = -23.26, *p* <.001, BCa 95% CI [-12.12, -10.23], η_p_² = 0.56).

Differences between the two groups at follow-up were found on the catastrophic sub-scale (F(1,428) = 342.18, *p* <.001, η_p_² = 0.44). The experimental group exhibited significantly lower scores on the catastrophic sub-scale (Adj. mean = 3.98, BCa 95% CI [3.49, 4.50]) compared to the control group (Adj. mean = 10.58, BCa 95% CI [10.01, 11.12]). The mean difference between the experimental and control groups was statistically significant at -6.59 (SE = 0.36, t = -18.53, *p* <.001, BCa 95% CI [-7.29, -5.89], η_p_² = 0.44).

Consistently across all in the three sub-scales, the experimental group exhibited lower scores compared to the control group (see Table 2).

### Differences between Baseline and Follow-Up in the Experimental Group

Patients attended an average of 3.2 treatment sessions (SD = 1.5), with no instances of missed sessions. Table 3 shows differences between baseline and follow-up in psycho-physiological parameters for the experimental group. Statistically significant reductions in psycho-physiological parameters were noted for all average values at the follow-up compared to the baseline assessment. The results show that there was a weak negative correlation between the number of biofeedback sessions and the respiratory rate (number of breaths per minute) and elevations respiratory rate (number of breaths per minute). The higher the number of sessions, the lower the respiratory rate and the elevations respiratory rate (r_S_ = − 0.201, *p* =.003 and r_S_ = − 0.173, *p* =.011 respectively).

**Table 3:** Differences in psychophysiological parameters within experimental group (*N* = 217)

Psychophysiological parameters	Baseline Mean (SD)	Follow-up Mean (SD)	Statistical test*	*p* value	Effect size
EMG (µV) Elevations EMG (µV) Respiratory rate (number of breaths per minute) Elevations Respiratory rate (number of breaths per minute) BVP (bpm) Elevations BVP (bpm) Skin conductance (µS)	68.0 (133.4) 73.1 (139.0) 13.1 (1.1) 19.7 (0.8) 81.1 (14.5) 85.7 (15.0) 2.0 (8.5)	8.6 (21.6) 8.7 (21.0) 11.2 (2.3) 13.0 (8.3) 71.5 (10.7) 73.3 (10.4) 1.0 (0.8)	1.607 920.5 2.555 218 2.523 1.448 704.9	**0.000** **0.000** **0.000** **0.000** **0.000** **0.000** **< 0.001**	0.5 0.6 0.4 0.7 0.4 0.5 0.06

Note. *Wilcoxon

## Discussion

This study aimed to assess the therapeutic effectiveness of a brief biofeedback treatment for tinnitus, integrated with breathing and relaxation exercises, within the routine of a medical center, with a focus on reducing daily living disability over a 3-month follow-up period. The main findings indicate a significant reduction in symptoms severity among patients in the experimental group compared to those receiving treatment as usual.

The symptoms reduction was assessed through the differences between the two groups in THI scores.

While it is conceivable that the differences between the two groups at follow-up were due to the biofeedback treatment, breathing exercises, and relaxation training, it is also possible that the improvement observed in the experimental group was influenced by a higher number of visits and the presence of a supportive therapist who listened carefully to patients and provided valuable information about tinnitus. Moreover, the patients in the control group were taking a greater number of medications compared to the experimental group. The higher number of medications could be considered indicative of greater symptom severity in these patients. For this reason, the difference observed at follow-up between the two groups should be interpreted with caution because the patients in the experimental group might have been less severe. However, this hypothesis actually contrasts with what was reported by the patients regarding their perceived tinnitus handicap severity. In fact, there were no differences between the two groups at baseline in THI scores, except for the functional sub-scale. Both groups exhibited a high degree of self-perceived disability at baseline, reflecting the significant impact of tinnitus on their daily lives. This impact was particularly evident in terms of affective responses to tinnitus and reactions such as loss of control, inability to escape from tinnitus, or effectively cope with it. After the 3-month follow-up, a statistically significant distinction between the two groups emerges: the experimental group exhibits lower THI scores, suggesting a reduction in impairment compared to the control group. Specifically, the experimental group’s scores reflect a mild level of tinnitus that is easily masked by environmental sounds and conveniently forgotten during activities. Although it may occasionally interfere with sleep, it doesn’t hinder daily activities (Schecklmann et al., 2015). In contrast, the control group reports a moderate level of tinnitus, which may still be noticeable even in the presence of background noise, yet it does not significantly impact daily activities.

This intervention’s impact is further underscored when examining the THI sub-scales. Our analysis reveals differences between the two groups at follow-up in the functional sub-scale. This sub-scale gauges role limitations in mental functioning, such as concentration difficulties, as well as in social and occupational spheres, reflecting the influence of tinnitus in social and relational activities (Newman et al., 1996). Patients in the experimental group showed an improvement in addressing these functional limitations, attributed to patients adopting strategies involving learning, knowledge acquisition, and symptom tolerance (Heinecke et al., 2009). Moreover, our investigation reveals a reduction in the emotional sub-scale within the experimental group. This sub-scale encompasses affective responses, including anger, frustration, irritability, and depression. Lastly, patients in the experimental group reported a decrease in the catastrophic sub-scale. This component encapsulates feelings of desperation, a perceived inability to escape from tinnitus, and a sense of being afflicted by an overwhelming ailment (Newman et al., 1996).

These results suggest that the benefits observed in the experimental group stem from patients’ ability to regulate physiological parameters such as respiratory frequency, heart rate, and muscle tension. This regulation contributes to improvements in the functional, emotional, and catastrophic components associated with tinnitus. Researchers have proposed three levels of tinnitus expression: physiological, organic, and emotional (Sereda & Hoare, 2015; Van de Heyning et al., 2015). Physiological tinnitus is universally experienced and is linked to the perception of internal and external sounds. Organic tinnitus, on the other hand, is associated with organic ear-related causes and often involves heightened sound perception compared to physiological tinnitus. The concept of emotional tinnitus, proposed by Hallam et al. (1988), emphasizes that the perceived severity of tinnitus does not strongly correlate with clinically assessed loudness. This implies that the level of disability and suffering is not solely determined by the level of dysfunction. Importantly, psychological factors play a crucial role in the manifestation of tinnitus symptoms (Hallam et al., 1988). These factors encompass not only stress and anxiety levels but also psychological causes. It is noteworthy that the study was conducted during the Covid-19 pandemic. Covid-19 and the resulting restrictions likely represented significant stressors for the subjects involved in the study, which may have influenced the onset, maintenance or worsening of symptoms. By incorporating breathing training and muscle relaxation, biofeedback has the potential to foster habituation and alleviate emotional tinnitus (Weise et al., 2008). Thus, heightened awareness of tinnitus can amplify perceived suffering and vice versa. A reduction in distress and autonomic system arousal can prove beneficial in mitigating symptoms. Notably, emotional tinnitus, activates attention and focusing mechanisms that pose challenges in tolerating the symptom. In this regard, biofeedback leverages body awareness to assist patients in managing symptoms by redirecting their attention focus (Kirsch et al., 1987). Between sessions, patients were given breathing and muscle relaxation homework. It is possible that these activities helped patients reduce distress. However, this should be interpreted cautiously because we did not collect data on the number or duration of these practice activities performed outside the clinic.

Significant differences in psycho-physiological parameters between baseline and follow-up could provide additional support for the efficacy of biofeedback intervention, relaxation training and breathing exercises among the treated group. It cannot be stated with certainty that these differences are a result of the intervention, as patients in the experimental group may have been able to control some potentially important psycho-physiological processes at follow-up. However, reductions in muscle tension, respiratory rate, heart rate, and skin conductance were observed, echoing previous studies (Khazan, 2018; Shaffer & Meehan, 2020). This study also reveals reductions in the elevated respiratory rate and heart rate. Patients suffering from tinnitus often exhibit heightened muscle tone, rigidity, and an increase in the temporomandibular joint muscles (Palmer & Durham, 2021; Whyte et al., 2021). Additionally, they experience sympathetic nervous system activation through hyperventilation. Therefore, addressing muscle rigidity directly through biofeedback proved instrumental in controlling symptomatology. Hallam et al. (1988) posit that tinnitus distress arises from heightened patient attention towards the tinnitus sound itself. The concept of somatic attention underscores how decreases in external stimuli intensity can elevate the salience of internal information. Prior research has indicated that relaxation can positively affect stress and discomfort experienced by those with subjective tinnitus (Jakes et al., 1986; Lindberg et al., 1987). Biofeedback, as a treatment modality, facilitates the voluntary control of autonomous or central nervous physiological parameters through operant conditioning. Specific variables like skin conductance and heart rate are displayed in real time, allowing autonomous function modification through learning.

This study also indicates favorable outcomes concerning the number of sessions required to observe an effect. The initial sessions focus on addressing hyperventilation and respiratory regularization. Each biofeedback session aims to facilitate self-regulation of breathing and muscle tension through relaxation procedures. Since patients with tinnitus experience both respiratory deficits due to hyperventilation and incorrect postures due to muscle rigidity, the training empowers them to regulate respiratory rate and identify triggers for respiratory activation. Muscle relaxation exercises lead to improvements in tinnitus function through guided exercises for the head, shoulders, face, and temporomandibular joint. By allowing patients to monitor their movements and muscle tension in real time, biofeedback empowers them to achieve greater control over physiological parameters. This approach rests on the premise that individuals can learn to control physiological parameters that they aren’t usually aware of if they receive feedback. Ultimately, the ability to reduce muscular tension coincides with a decrease in EMG activity, resulting in a return sound signal that decreases in frequency. Consequently, patients are guided towards relaxation and self-control through subsequent tests (Biondi & Valentini, 2014). Patients recognized the association between body indices (e.g., heart rate, muscle tension, electrodermal activity, etc.) and their internal state. This recognition facilitates the voluntary control of physiological states by reducing sympathetic activity and increasing the parasympathetic. As a result, enhancing respiratory and muscular functionality not only mitigates tinnitus symptoms but also enables patients to divert their attention from the disorder. Engaging in distracting activities reduces the frequency of brooding processes. Importantly, both visual and auditory feedback have been used to control physiological parameter variability, without aiming to mask the tinnitus sound itself.

### Limitations

While this study offers valuable insights, several limitations warrant consideration. Firstly, by relying only the four measures of the THI, we may have overestimated the effects. Besides, there is the issue of recruitment process: the absence of randomization may have introduced selection biases, impacting the study results and generalizability. The consecutively recruited subjects may not be representative of the target population. Furthermore, the study’s scope was confined to a single medical center in the northern Italy, potentially limiting the broader applicability of the findings. Another noteworthy limitation pertains to the lack of information concerning patient personality traits or other features that could enhance sample homogeneity. For the biofeedback protocol, an initial and final two-minute baseline was employed. Studies differ on the optimal duration of baseline measurements. Harstrup (1986) highlighted a lack of consensus regarding baseline duration, while Gerin et al. (1994) found that a one-minute baseline may be sufficient for heart rate stabilization. However, as most validated protocols for stress treatment use a two-minute baseline (Khazan et al., 2009; Cram et al., 1999; Schwartz et al., 2017), we chose to follow this standard procedure.

Additionally, other mechanisms, such as meeting a therapist who listens to and supports the patient, or receiving useful information for managing tinnitus, may have played an important role in the changes observed in the experimental group. Last but not least, it remains uncertain whether the control group experienced any alterations in the psycho-physiological parameters monitored in the experimental group, as this aspect was not explored. Moreover, as psycho-physiological parameters were not collected in the control group, it remains uncertain whether adaptation and habituation contributed to the observed changes, and to what degree these changes were influenced by the experimental treatment.

## Conclusions

This study highlights the effectiveness of using brief biofeedback training, combined with breathing and relaxation exercises, in treating tinnitus patients within a medical center setting. By disrupting the stress-induced vicious cycles that underlie tinnitus etiology, this non-pharmacological intervention has demonstrably enhanced patients’ quality of life. Tinnitus often inflicts impairment on patients’ daily existence, affecting work, relationships, and sleep, while also triggering an attentive fixation on the symptom itself. Through biofeedback, along with breathing exercises and relaxation training, patients learned strategies for managing symptoms, using muscle relaxation and respiratory control to reduce the anxiety, arousal, and heightened alertness caused by tinnitus. Future investigations are warranted to delve deeper into the mechanisms underlying tinnitus pathology and to explore potential psychological and personality factors that could contribute to the onset of tinnitus symptoms.

## Data Availability

Data will be made available on request.
